# Extended Conformational Selection in the Antigen–Antibody Interaction of the PfAMA1 Protein

**DOI:** 10.1021/acs.jpcb.4c03734

**Published:** 2024-08-22

**Authors:** Pamella Cristiny Carneiro da Silva, Leandro Martinez

**Affiliations:** Institute of Chemistry and Center for Computing in Engineering & Sciences, Universidade Estadual de Campinas (UNICAMP), 13083-861 Campinas, SP, Brazil

## Abstract

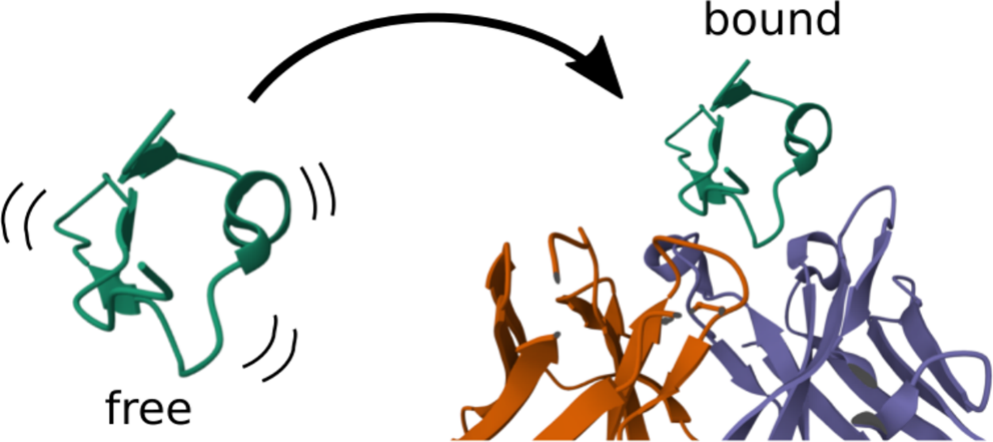

*Plasmodium falciparum* apical membrane antigen 1 (PfAMA1) is a surface protein found in two stages of the malaria life cycle. This is a protein involved in a reorientation movement of the parasite so that cell invasion occurs in the so-called “moving junction”, relevant when the membranes of the parasite and the host are in contact. The structure of a conformational epitope of domain III of PfAMA1 in complex with the monoclonal antibody Fab F8.12.19 is experimentally known. Here, we used molecular dynamics with enhanced sampling by Hamiltonian replica exchange molecular dynamics (HREMD) to understand the effect of intermolecular interactions, conformational variability, and intrinsically disordered regions on the mechanism of antigen–antibody interaction. Clustering methods and the analysis of conformational variability were used in order to understand the influence of the presence of the partner protein in the complex. The free-state epitope accesses a broader conformational pool, including disordered conformations not seen in the bound state. The simulations suggest an extended conformational selection mechanism in which the antibody stabilizes a conformational set of the epitope existing in the free state. The stabilization of the active conformation occurs mainly through hydrogen bonds: Tyr(H33)-Asp493, His(L94)-Val510, Ser(L93)-Glu511, Tyr(H56)-Asp485, and Tyr(H35)-Asp493. The antibody has a structure with few flexible regions, and only the complementarity determining region (CDR) H3 shows greater plasticity in the presence of the epitope.

## Introduction

In protein–protein interactions (PPIs), the conformation of a protein acts as an environment, or a set of preconditions, for the conformations of the partner protein.^1^ The mechanisms of protein–protein association can involve multiple steps with variable energetic barriers. Each mechanism has its functional importance, and insights into the structure of the proteins involved can be obtained by experimental studies using X-ray crystallography, cryoelectron microscopy, chemical kinetics, and nuclear magnetic resonance.^2^ The binding events are not necessarily well demarcated, but they can be understood and classified in general terms, and reveal the importance of specific conformations and the protein environment in a PPI. The description of biomolecular recognition is important for the development of therapeutics, as it covers PPI, protein–ligand, and substrate interactions from the signaling stages to cellular metabolism.^3^

The PPI mechanisms can vary, particularly when disordered proteins are involved. The mechanisms of protein–protein association can be roughly classified into the lock-and-key, induced-fit, or conformational selection models. In the lock-and-key model, the conformations of the proteins before association are similar to those after association, and binding depends on their attachment with proper relative orientations. In the induced-fit model the conformations assumed by the binding partners are not populated in the unbound state, and are assumed throughout the binding process as a result of the local interactions. In the conformational selection model, on the other hand, conformations necessary for binding exist in the free state. In many cases, these mechanisms cannot be clearly distinguished, and are considered cases of “extended conformational selection mechanisms”.^4^ Accordingly, conformational selection can be followed by induced fit and mutual adjustments between participating components.^5^

*Plasmodium falciparum* (*Pf*) apical membrane antigen 1 (PfAMA1) is a surface protein found in two stages of the malaria life cycle. It is a relevant target for the development of effective vaccines and treatments. AMA1 is well conserved among *Plasmodium* spp. Its structure is rich in cysteine, and thus its scaffold is determined by intramolecular disulfide bonds.^6^ This protein is found in the merozoite and sporozoite stages of *Pf*, during the invasion of liver and blood cells, respectively. Its function is involved in the so-called “moving junction”, that is formed when the membrane of the parasite and the host are in close contact to enable invasion. Adhesion of the parasite to human cells is followed by a reorientation movement of the merozoite and sporozoite necessary for the apical pole to come into contact with the surfaces of the hepatocyte orerythrocyte cells.^7^

Studies show that different domains of AMA1 are capable of producing immunological responses. A possibility to overcome the challenges to finding effective treatment imposed by the malaria life cycle are recombinant multiallele proteins that have joint action in structured and disordered regions. The affinity of purified human antibodies against PfAMA1 domain III epitopes inhibited parasite invasion,^8^ and synthetic peptides that mimic the loop region of domain III were used as immunogens to generate monoclonal antibodies.^9^ A high level of parasite growth inhibition was observed, around 95%. These studies suggest that the molecular understanding of the antigen–antibody interactions, including the role of conformational plasticity of the patterns, can help the rational development of synthetic peptides for the inhibition of the malaria life-cycle.

In this paper, we perform enhanced sampling molecular dynamics simulations to study the plasticity and the mechanisms of conformational stabilization of domain III of PfAMA1 with the fragment antigen-binding region (Fab F8.12.19) of the monoclonal antibody. We provide insights into the antigen–antibody regions and conformational changes associated with the development of antimalarial mechanisms.

## Methods

Here, we used the crystallographic model obtained by Igonet and collaborators^10^ of a complex of an epitope in domain III of the AMA1 protein, and the Fab region of the recombinant monoclonal antibody F8.12.19 (PDB ID:2J5L). The Fab F8.12.19 recognizes the conformational epitope located in domain III, indicated as an antigenic region against antibodies.

The content of disorder of the epitope was analyzed with DISOPRED3^11^ and with the RAPID web server (http://biomine.cs.vcu.edu/servers/RAPID/).^12^ The molecular dynamics (MD) of the PDB structures were performed for three systems:

(1) Isolated epitope of the 2J5L structure in water.
(2) Free antibody in water.
(3) Antibody–antigen complex in water.

The solvated boxes were generated using Packmol.^13^ GROMACS 2019.4^14^ with the PLUMED^15^ interface was used to perform Hamiltonian replica exchange molecular dynamics simulations (HREMD).^16^ The CHARMM36m force field was applied for the protein, and the TIP3P model for water.^17^

In HREMD simulations, multiple replicas of the system are simulated in parallel, and a potential energy perturbation (usually smoothing) is applied to a subset of the systems in progressive fashion. The perturbation is designed to accelerate the motions of the parts of the systems of interest, feeding the nonperturbed replica with a greater conformational sampling by means of a Monte Carlo exchange probability. The conformations obtained for the nonperturbed replica follow the correct thermodynamic weights, and are used to study the conformational ensemble of the protein regions of interest.

Test simulations varying the range of perturbations were conducted with a Hamiltonian scaling factor λ ranging from 0.6 to 1.0 aiming an exchange rate of 20 to 40%, as recommended in the literature.^18^ Here, λ = 1.0 refers to the unperturbed replicas. Finally, HREMD simulations with 10 replicas, for each of the 3 systems, with λ ≅ 0.71 to 1.0, with steps of 0.029,^19^ allowed exchange rates of ∼30% to be achieved for all systems.^20^

This perturbation range was selected to perform the 10 replicas of 500 ns HREMD simulations, totaling 5 μs of simulation for each system. In system 1, scaling was applied across the entire epitope. In system 2 the scaling factor was applied only to the antibody antigenic site loops, the complementarity-determining regions (CDRs). Here, the six loops that form the CDRs are identified as L1, L2, and L3 for the light chain and H1, H2, and H3 for loops formed by the heavy chain. In the hydrogen bonds, H and L are also used to identify residues that are part of the antibody chains as well. In system 3, the two individual scalings were combined: enhanced sampling was performed on the full epitope and on the antibody CDRs.

The conformational variability of the proteins was studied using the root mean square deviations (RMSD) and the root mean square fluctuations (RMSF), calculated using the MDLovoFit^21^ software. MDLovoFit identifies rigid and mobile substructures automatically,^22^ providing a rich view of the stable and flexible parts of the structures. With this classification, the RMSD can be understood in terms of global fluctuations of the structure, or the divergent movement of some structural subgroups. Radius of gyration (R_G_) and the solvent accessible surface area (SASA) were computed with GROMACS functions, *gmx gyrate* and *gmx sasa*, respectively. These are also properties associated with the magnitude of conformational changes induced by PPI. Here, we report the relative SASA (S_rel_) for free and bound states of the molecule,^23^ and the reference structures for the calculation of Srel were the crystallographic structure (2J5L). When correlated to the RMSD, the Srel provides a two-dimensional characterization of the protein structural changes in the simulation. To map the similarity of the conformations in the trajectories we used hierarchical clustering analysis (HCA) with the TTClust software^24^ and the Ward algorithm.^25^ The clustering was based on the matrix of distances of RMSDs. The distances between secondary structure motifs were defined by closest α carbon atoms (Cα) in the crystallographic model. The distance assumed between the two β-sheets is Met496/Cα-Phe505/Cα, and the distance between the β-sheet and the α-helix is Phe505/Cα-Lys485/Cα. Structure figures were produced with VMD.^26^

## Results and Discussion

### Protein Flexibility of the *Pf*AMA1 Epitope

**Figure 1:**
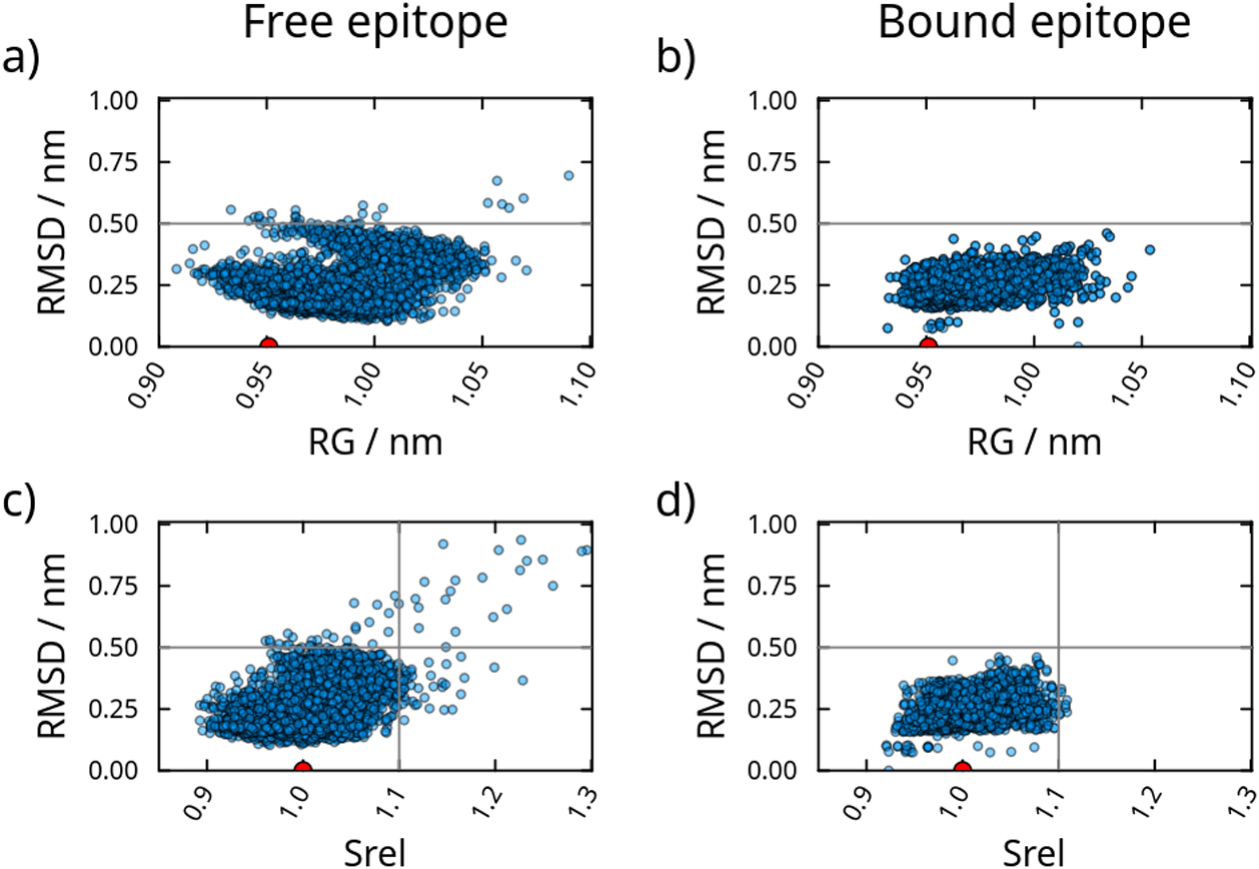
Conformations obtained, represented by RMSDs as a function of the radius of gyration (*R*_G_) or relative solvent accessible surface area (*S*_rel_). The free epitope in solution is shown in (a, c), and the bound epitope is shown in (b, d). The crystallographic structure has an *R*_G_ of 0.951, and was used as the reference state for the computation of the RMSDs and *S*_rel_. The red dot indicates the position of the crystallographic model in the plots.

The DISOPRED3 analysis indicated that the AMA1 epitope contributes with 10% of disorder in its structure. In order to understand whether this degree of disorder is sufficiently relevant to the functional conformation of the epitope, the RMSD as a function of the radius of gyration (*R*_G_) and of relative solvent accessible surface areas (*S*_rel_) were analyzed. Figure 1 shows the structures displaying each combination of these structural features in the conformations obtained. The free epitope displays a greater conformational plasticity than the epitope bound to the antigen. Although structures with RMSDs close to the crystallographic pose were frequently sampled (RMSDs within ∼0.2 and 0.3 nm) in both states, regions of the conformational space with higher values of RMSD and *R*_G_ were observed (above ∼0.5 nm) in the free form (Figure 1a,b). Similarly, the free epitope accessed structures with greater solvent accessible surface area, correlated with the greater RMSDs, as shown in Figure 1c,d.

A multidimensional visualization of protein flexibility is possible from the solvent accessible surface areas. This accessibility is particularly important, as it has been associated with the antigenicity of epitopes.^27^ The free epitope presents disordered conformational structures with *S*_rel_ >1.1 (Figure 1c). Nevertheless, the largest set (*S*_rel_ ∼1.0) corresponds to the RMSDs of the packed structures (∼0.2 nm), with a smaller surface area. A set of partially unstructured epitopes is also present with RMSD close to 0.5 nm and *S*_rel_ 1.1. The epitope in the bound state displays structures with low *S*_rel_ values, indicating that this conformational set has areas with variations close to those of the crystallographic structure (Figure 1d), a compact structure commonly observed in conformational epitopes that interact with discontinuous amino acid residues.^28^ This supports the functional relevance of the conformational set close to the more compact structures, including the crystallographic pose, with lower *S*_rel_ and RMSD values. These results corroborate experimental studies that show that although *Pf* surface proteins have high degrees of disorder, the stability brought by AMA1’s disulfide bonds is determining for its functional role.

**Figure 2:**
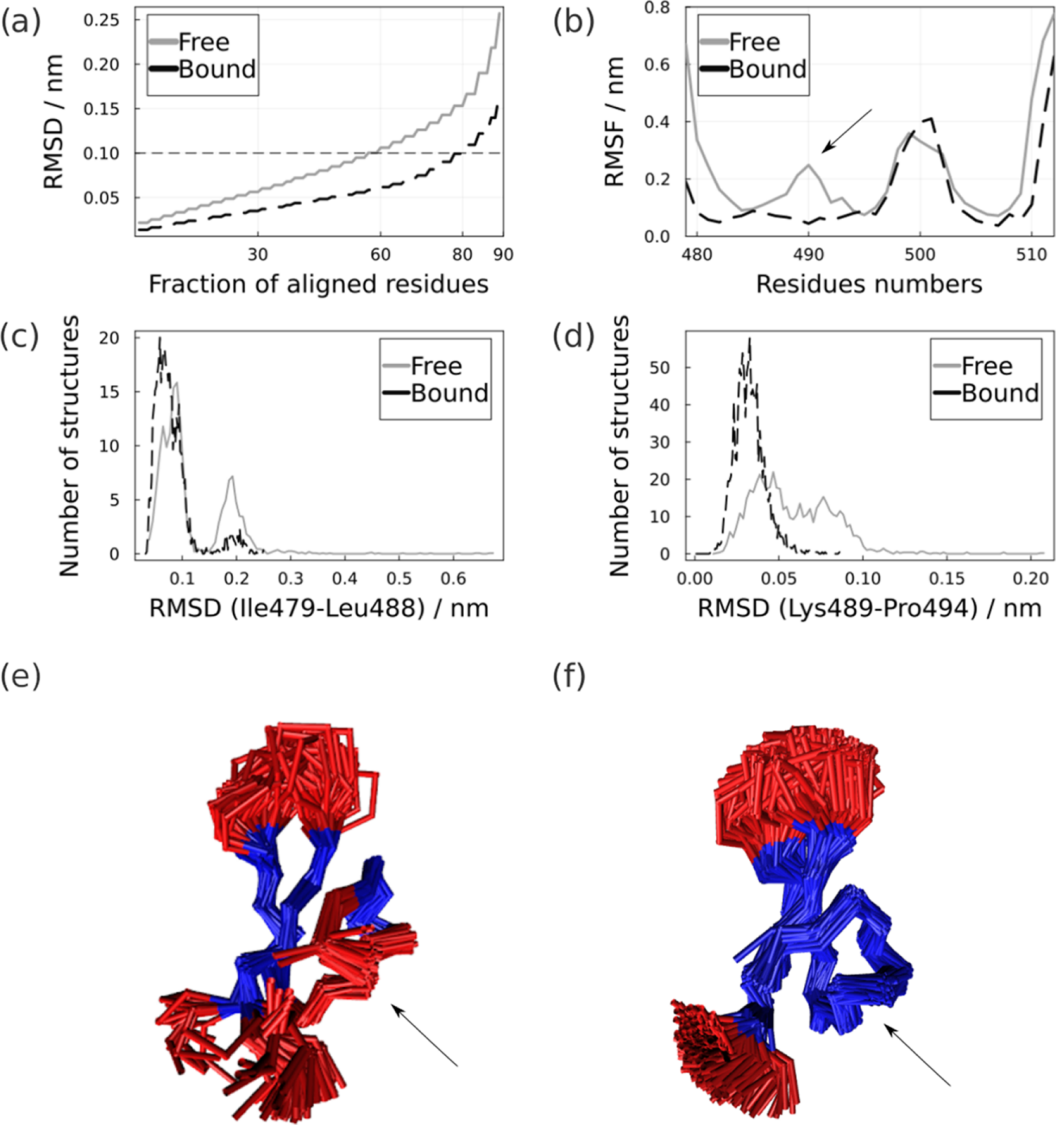
Visualization of epitope flexibility in free and bound states. (a) Fractions of aligned residues in function of RMSD and (b) RMSFs for the free (gray) and bound (black) epitopes. Density of the RMSDs values for (c) Ile479-Leu488 and (d) Lys489-Pro494 regions in both states. Flexible (red) and rigid (blue) structural subsets of the epitope in free and bound states are shown in (e, f). The region of interaction with antibodies (Lys489-Pro494) is indicated by the arrow.

Figure 2 shows protein structural fluctuations as analyzed with MDLovoFit. Figure 2a shows that in the free state, 60% of the residues can be aligned with structural deviations smaller than 0.1 nm (gray), while 80% of the residues display the same level of structural rigidity in the bound state (black). Thus, while these results confirm the presence of a rigid epitope core, binding to the antigen promotes a significant stabilization of regions that are flexible in the free form.

Figure 2b–d illustrate the position in the sequence and structure of the different protein flexibilities. The region that becomes more structured can be seen in the RMSF plot (Figure 2b), in which there is a decrease in the fluctuation peak of residues with numbers close to 480 (479 to 488) and 490 (489 to 494). These residues correspond to the C-terminal region close to the α-helix and the loop of the interaction with the antibody, which are disordered regions of AMA1, and can be visualized in Figure 2c,d.

Figure 2c,d shows the distribution of RMSDs of the intrinsically disordered region (IDR) that interacts with the antibody (Lys489-Pro494) and the region of the α-helix (Ile479-Leu488) close to the C-terminal region in the crystallographic structure, which are the epitope regions that became more structured with the interaction, as seen in Figure 2b. These two regions in the free epitope present a distribution with more dispersed values and even greater than 0.5 and 2 nm, respectively. Indicating structures in which these regions contribute to more dissimilar conformations. While in the bound state, the RMSD values are in a range that does not differ greatly from the crystallographic pose. This reinforces the observations above that the interaction with the antibody provided a structured behavior for these regions in the complex.

### Conformational Sets

**Figure 3:**
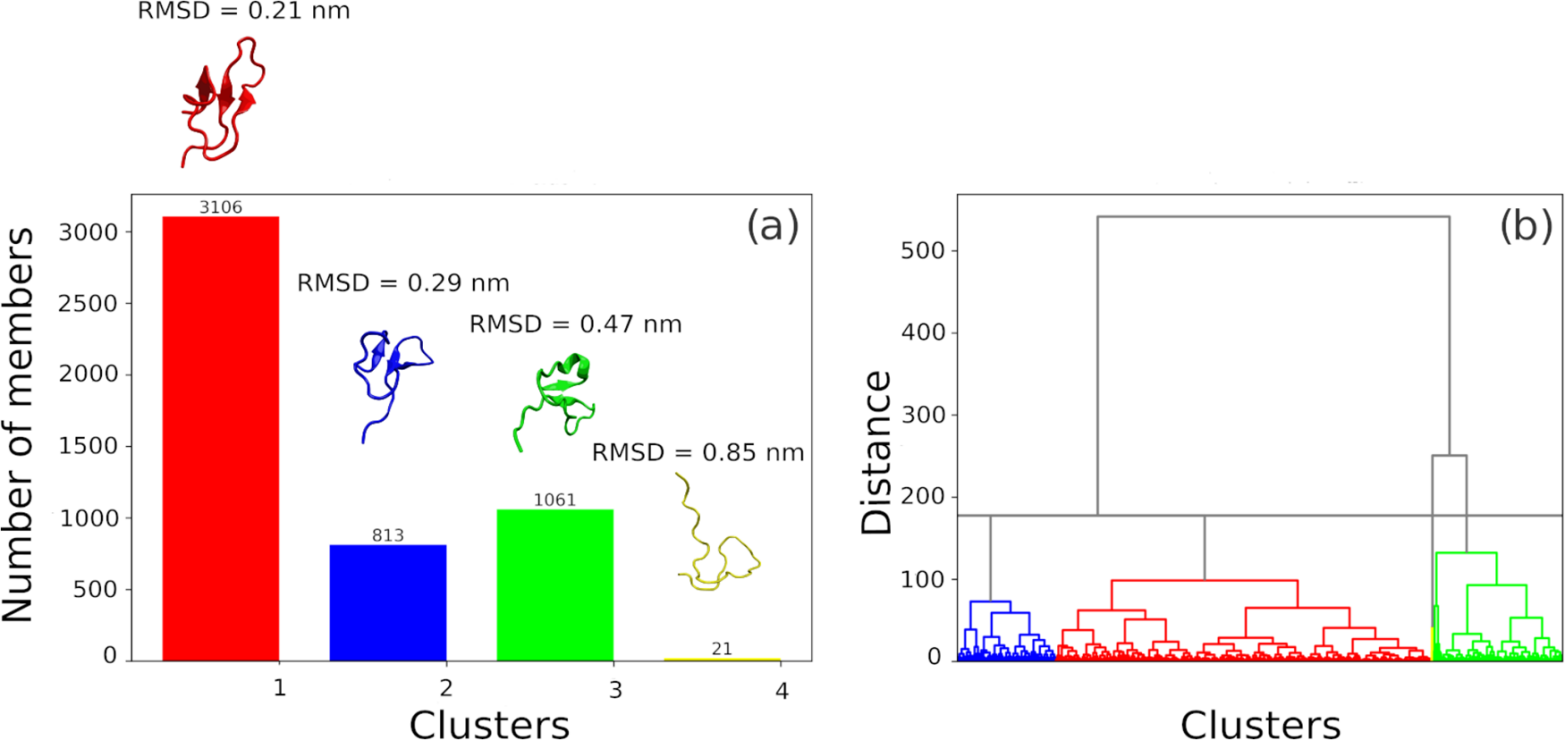
Clustering of structures obtained in the free epitope trajectory by HCA (a) number of structures per cluster and (b) dendrogram.

The HCA analysis of structure similarity by RMSD (Figure 3) shows four structural sets that are most dissimilar to each other. The red cluster corresponds to the most populated structures in the simulation, in which the epitope remains ordered (RMSD = 0.21 nm of the most representative structures in relation to the crystallographic pose). There are still partially unstructured states, corresponding to the blue (RMSD = 0.29 nm) and green clusters (RMSD = 0.47 nm), and the epitope accesses completely disordered conformations on a smaller scale (yellow cluster) with the greatest dissimilarity (0.85 nm).

**Figure 4:**
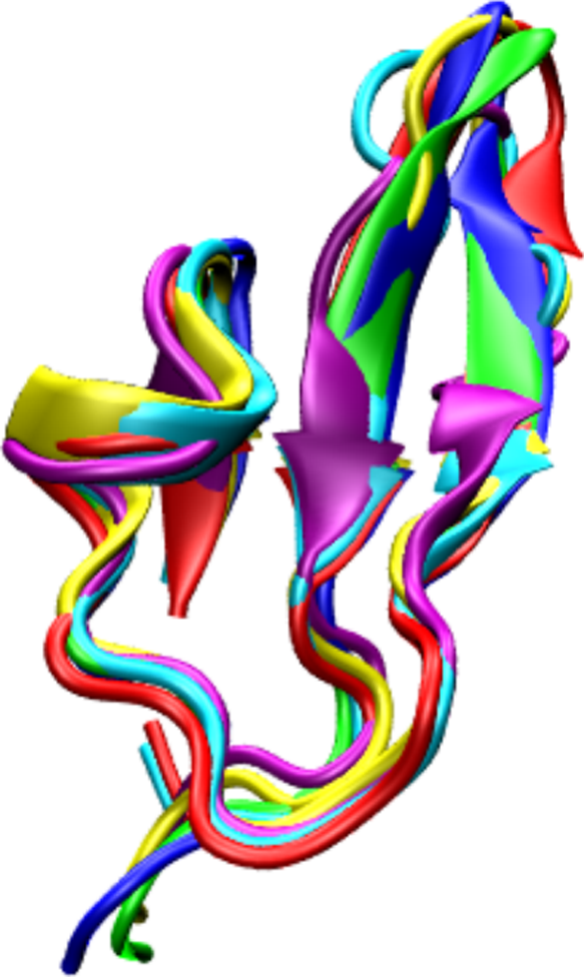
Alignment of the most representative structures of the cluster groups for the bound epitope. This alignment of the structures in the bound state does not have major variations in relation to the red cluster in Figure 3.

The alignment of the clustered structures of the epitope in the bound state (Figure 4) shows that the most representative structure is very close to the red cluster of the epitope in the free state. Also, there is no great variation in conformations in the bound state. The RMSDs of these bound structures in relation to the crystallographic structure have a minimum of 0.1 nm and a maximum of 0.26 nm. This may indicate that the interaction occurred with the most likely conformation of the molecules before the formation of the complex and the presence of the partner protein contributed to increasing the structuring of the PfAMA1 conformational epitope. The stability of the set of conformations in regions close to an active pose (in this case, the crystallographic conformation) is an indication of conformational selection in the state before the interaction.^27^

### Tertiary Structure

**Figure 5:**
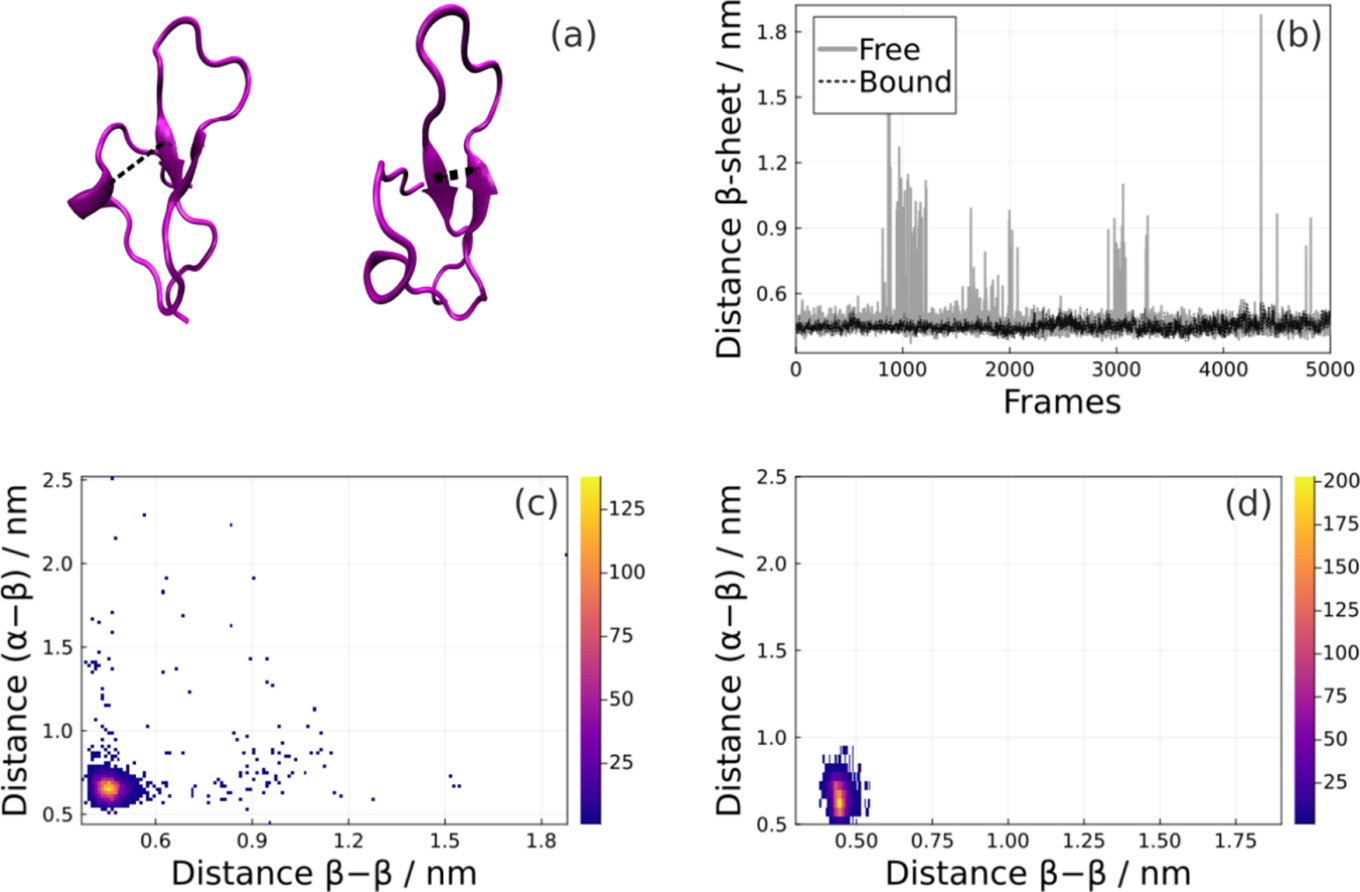
(a) Distance between α-helix and β-sheets. (b) β-sheets distances along the MD in both states, and separation between the α-helix and the β-sheets in the (c) free and (d) bound states.

The distance between the secondary structure motifs also helps to characterize the packaging of the tertiary structure of the epitope. Figure 5a shows the distances assumed between the two β-sheets (defined as the distance between Met496/Cα and Phe505/Cα) along the trajectory, and the distance between the β-sheet and the α-helix (Phe505/Cα-Lys485/Cα). These observations are useful for understanding how these secondary structures remain stabilized among the observed conformations. In both states, the two β-sheets remain very close, except when the epitope assumes disordered conformations in the free state (Figure 5b).

The α-helix has a movement in relation to the β-sheet structures, in which is observed an unfolding of this epitope in smaller portions of the space in the free state (Figure 5c). In its functional state, it remains stabilized close to the β-sheets throughout the trajectory (Figure 5d). This corroborates the previous analyses that indicate a stabilization in this region in the presence of the antibody and the contribution of the region from Ile479 to Leu488 to the disordered structures observed in the noncomplexed trajectory.

These observations are important, as they may indicate that although the α-helix is a secondary structure present in the free state in which the epitope has greater mobility, it is not a fully stabilized region. Thus, the environment of the partner protein promoted the stabilization of the α-helix secondary structure, which in turn closely participates in the structuring of the tertiary conformation of the epitope. This is a common phenomenon in the process of protein–protein binding involving disordered structures.^29^

The induction of immune responses by PfAMA1 is related to the formation of intermolecular disulfide bonds that stabilize conformational epitopes.^6^ This characteristic has been well described in MD, since although the epitope has a reasonable set of conformations in the free state and is located in a disordered region of AMA1, the presence of disulfide bonds and secondary structures are more determining for its function than the regions of high flexibility, which were only accessed in a free state.

These analyses of the epitope in both states show that the interaction with the antibody was carried out with a conformation present in the free state, which was the most populated among the possible dynamic structures. The conformation compatible with the ligand is selected, and the entire conformational set is shifted to this state, favoring the stabilization of the secondary structure of the interaction site. Conformational selection was, therefore, the phenomenon observed with the decrease in RMSD and *S*_rel_ variations and the stabilization of secondary structures and IDR, the latter of which performs direct interaction in the antibody paratope. These observations are consistent with the functional relevance of the PfAMA1 conformational epitope in the literature.^4^

### Intermolecular Interactions and CDRs

**Figure 6:**
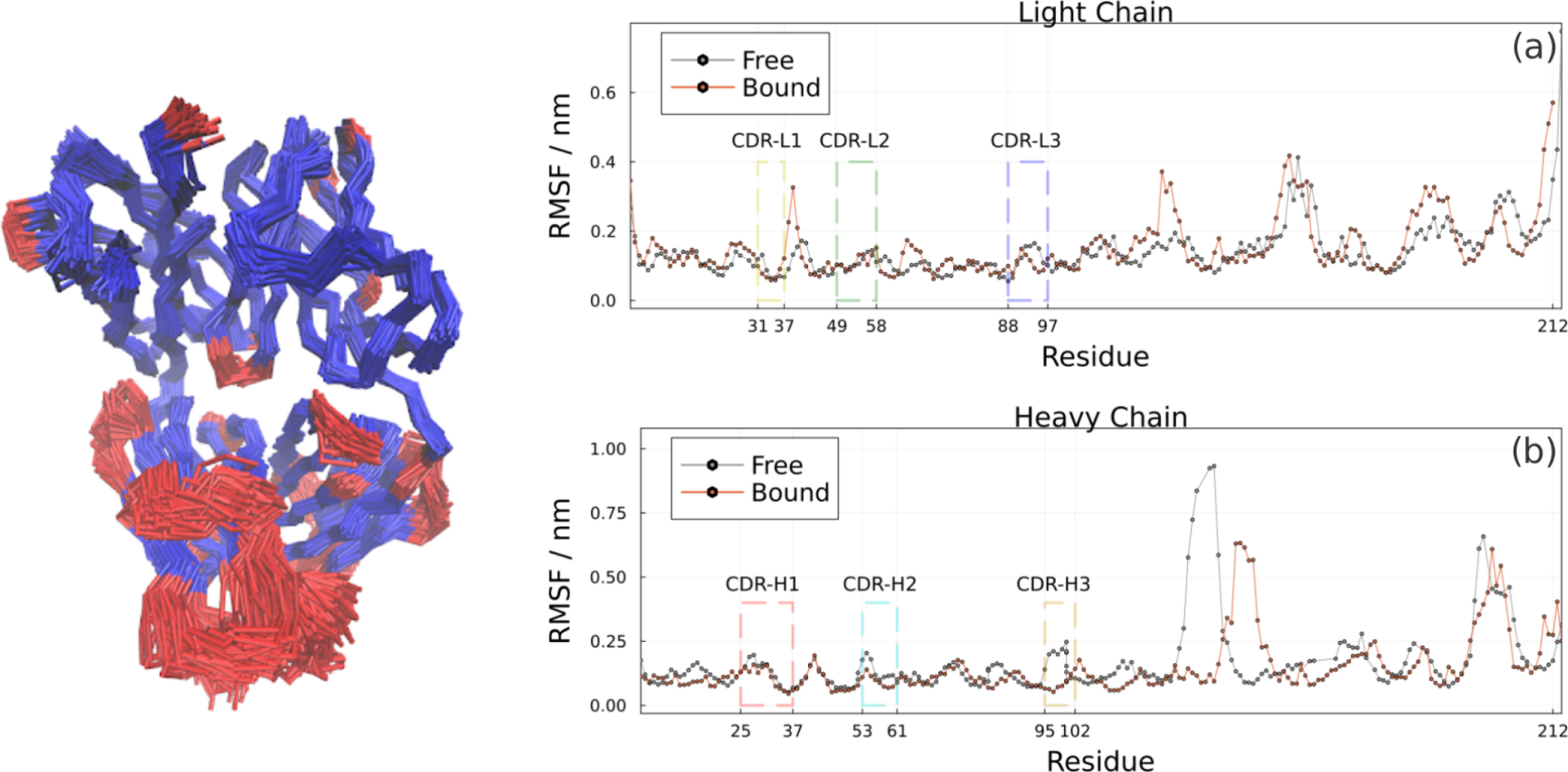
Flexibility of the Fab F8.12.19 antibody, as obtained with MDLovoFit. Conserved regions are in blue, and flexible regions are red. RMSFs of the (a) light (chain B) and (b) heavy chains (chain C) of the antibody.

**Figure 7:**
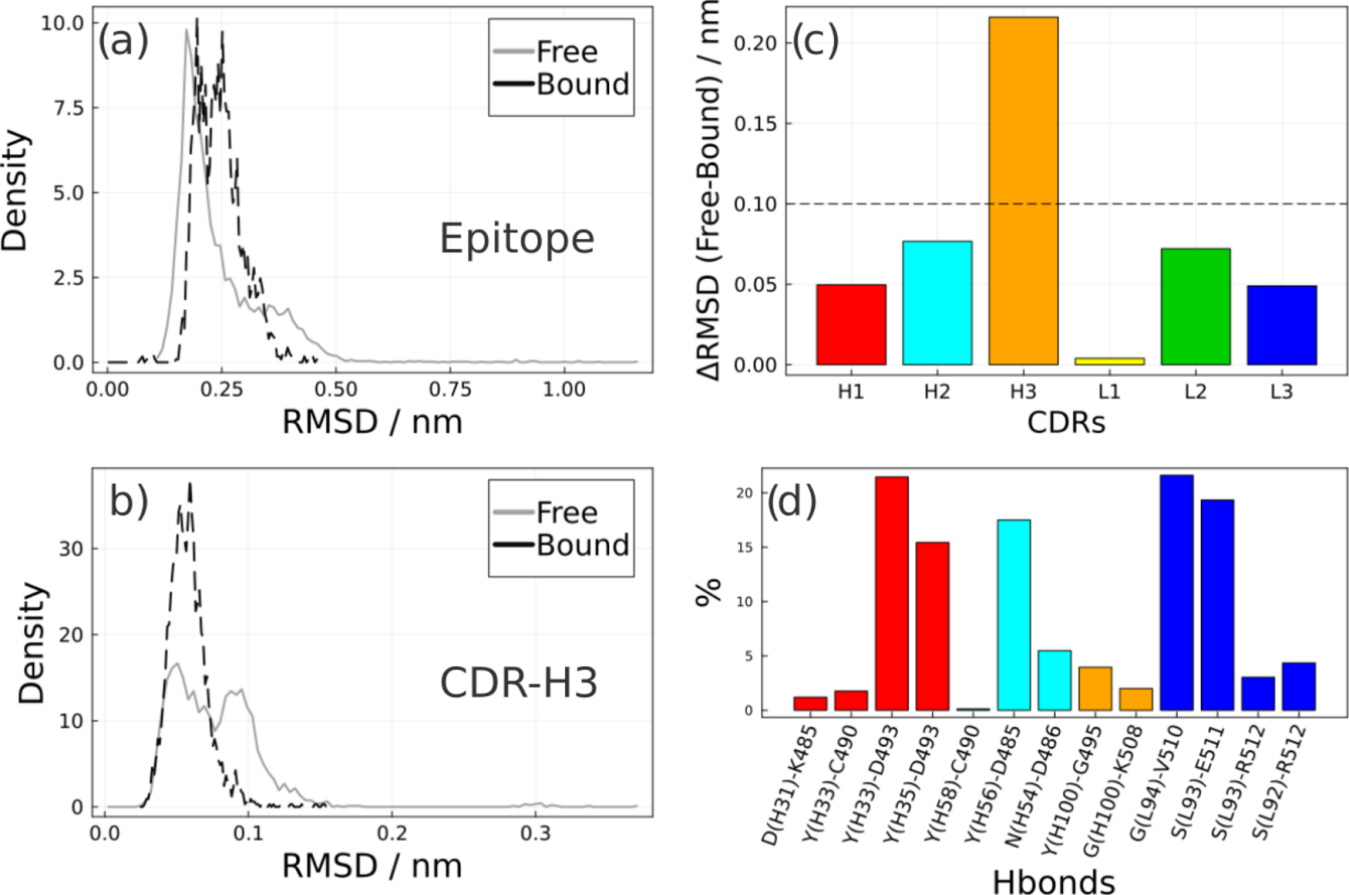
RMSDs of free and bound states of the (a) epitope and (b) CDR-H3 of the antibody Fab F8.12.19. (c) Differences in the largest RMSDs between the free and bound states of the CDRs of heavy chain (H1, H2, and H3 in red, cyan, and orange) and light chain (L1, L2, and L3 in yellow, green, and blue). (d) The percentage of conformations in the simulations in which hydrogen bonds between the epitope and the CDRs are observed.

The antibody paratope is formed by the heavy (H) and light (L) chains CDRs: CDR-H1 (25-SER to 37-VAL), CDR-H2 (53-GLY to 61-ASP), CDR-H3 (95-ASP to 102-TYR), CDR-L1 (31-SER to 37-GLN), CDR-L2 (49-HIS to 58-VAL), and CDR-L3 (88-CYS to 97-THR) (Residue numbering is according to Chothia et al.^30^) The monoclonal antibody has a structure with low flexibility. In MD results, in general, there are no major changes in the global structure of the bound and free states (Figure 6, in gray) taking into consideration that the sampling acceleration was applied only to the CDRs, which are the regions of antigenic interest and carry out specific interactions. In this sense, we observed some flexibility in the loops of the F8.12.19 Fab paratope (Figure 7c), and the most significant conformational variability was observed in CDR-H3.

The low flexibility in the paratope may to avoid entropic losses during the interaction with the antigen and provide a precise three-dimensionality presentation of key contacts, thus allowing a rapid association.^31^ CDRs play a fundamental role in antibody specificity and antigen recognition. Among the 6 loops that form the paratope, 5 adopt canonical conformations that are well characterized and facilitate computational work such as modeling and prediction.^32^ Studies^33,34^ show that CDR-H3 presents greater structural and conformational variability, even in comparison with different organisms, and may contribute to increased contacts.^35^ The RMSF graphs indicated that these characteristics were well described in MD, in which only CDR-H3 exhibited more appreciable plasticity with a decrease in RMSF in the presence of the epitope in the bound state. We can also say that there is still room for computational studies in the antibodies field, and a larger sample space will help us understand how these interactions happen at the molecular level.^36^

Comparing the graphs of the RMSD densities of CDR-H3 and the epitope in both states in relation to the crystallographic structure, the conformational variability of this interaction becomes clearer: The epitope (Figure 7a) and CDR-H3 (Figure 7b) present conformations that have more dispersed RMSD values only accessed in the free state. In the complex, the dispersion of CDR-H3 is more concentrated throughout the trajectory. The epitope in both states has a sampling concentration of RMSDs close to values up to 0.25 nm, which is still considered the reproduction of the binding mode of the crystallographic pose.^37^ The proximity of the overlap in the dispersions corroborates other analyses that, in the free state, the epitope presents conformations with RMSDs also observed in the bound state.

Figure 7d shows that the CDRs that had the most significant contributions to the stability of the complex were CDR-H1, CDR-H2, and CDR-L3. The most important interactions observed were: Tyr(H33)-Asp493, Tyr(H35)-Asp493, His (L94)-Val510, Ser(L93)-Glu511, and Tyr(H56)-Asp485. They were observed in greater proportions and remain stabilized throughout the trajectory. Among these interactions, Ser(L93)-Glu511 and Tyr(H56)-Asp485 were not reported in the crystallographic structure but are important to the complex as they appear frequently in the simulation. Another important observation is that the Fab F8.12.19 antibody was synthesized for the AMA1 species of *Plasmodium vivax* (PvAMA1), but it cross-reacted with PfAMA1. Some interactions present in the antibody with the PvAMA1 species were observed in the MD with the PfAMA1 epitope in smaller contributions: Asn(H54)-Asp486 and Ser(L92)-Arg512.

In the extended conformational selection mechanism described here between the PfAMA1 epitope and the Fab F8.12.19 monoclonal antibody, it can be understood that, in addition to the selection of the conformation of the epitope already existing in the free state, it induced a small adjustment in the antibody structure predominantly in the CDR-H3. The epitope presents reduced conformational variability when interacting with the antibody, which stabilizes the complex with hydrogen bonds. The functional epitope is structured and compact, and its bound conformational set has structures dynamically close to the crystallographic pose, corroborating the functional relevance of this conformation.

## Conclusions

Hamiltonian replica exchange molecular dynamics simulations were used to examine the dynamics of the PfAMA epitope in the free form and associated with the Fab antibody F8.12.19. The free-state PfAMA1 epitope accesses unstructured conformations, although displaying a larger population of conformations similar to the crystallographic conformation. This subset is selected and stabilized by complexation with the antibody.

The region of residues Ile479 to Leu488 has a great contribution to the mobility of the epitope. The α-helix in this region is destabilized when the epitope assumes partially or fully disordered conformations. Around 20% of the epitope undergoes stabilization when it associates with the antibody. Fundamentally, one of the stabilized regions presents disorder in the free epitope and is the main region of interaction with the paratope. Although the antibody interaction site is formed by loop regions, called CDRs, they do not present a large variation in flexibility between the free and bound states, and the greatest conformational variability was observed only in CDR-H3.

The most important hydrogen bonds observed were Tyr(H33)-Asp493, Tyr(H35)-Asp493, Tyr(H56)-Asp485, Ser(L93)-Glu511, and His(L94)-Val510 carried out by CDR-H1, CDR-H2, and CDR-L3. It was also possible to observe interactions that were reported only with the *P. vivax* species in the MD of this complex with Pf: Asn(H54)-Asp486 and Ser(L92)-Arg512. Other interactions that are frequently observed in the complex in molecular dynamics but do not appear in the crystallographic pose are Ser(L93)-Glu511 and Tyr(H56)-Asp485.

Computational methods are great allies to experimental methods to understand phenomena at a molecular level. It can be stated that the HREMD method was robust to describe the extended conformational selection interaction mechanism between the PfAMA1 epitope and the Fab monoclonal antibody F8.12.19. It was possible to characterize important interactions and understand the conformational variability of molecules in the presence or absence of their partner proteins. The study of antigen–antibody interaction is important due to the need to understand the stability, specificity, and antigenicity of *P. falciparum* proteins and regions of disorder for the development of effective antimalarial treatments.
